# The complete chloroplast genome of *Gardenia stenophylla* Merr (Rubiaceae) and its phylogenetic analysis

**DOI:** 10.1080/23802359.2024.2389918

**Published:** 2024-08-12

**Authors:** Shaoyong Deng, Cunzhong Fan, Ziyun Lu, Huilin Yang

**Affiliations:** a Jiangxi Academy of Forestry, Nanchang, China; b College of Life Sciences, Jiangxi Normal University, Nanchang, China

**Keywords:** *Gardenia stenophylla*, chloroplast genome, phylogeny

## Abstract

*Gardenia stenophylla* Merr, a member of the genus *Gardenia* in the family Rubiaceae, possesses significant medicinal and ornamental value and is widely distributed in China. This study reports the newly sequenced chloroplast genome of *Gardenia stenophylla* Merr. The complete chloroplast genome of *Gardenia stenophylla* Merr (155,109 bp, GC content of 37.5%) was shown to have a typical quadripartite structure, containing a pair of inverted repeat regions (IRs) of 28,802 bp separated by a large single-copy (LSC) region of 85,396 bp and a small single-copy (SSC) region of 18,109 bp. The chloroplast genome contained 151 genes encoding 106 proteins, 37 tRNAs, and eight rRNAs. The *Gardenia stenophylla* Merr chloroplast genome displayed the closest phylogenetic relationship to *Gardenia jasminoides* and *Gardenia jasminoides* var. grandiflora. These data will assist in future molecular phylogenetics of the Rubiaceae.

## Background

About 250 species of *Gardenia* are distributed in tropical and subtropical regions of the Eastern Hemisphere. There are only six species of *Gardenia* naturally distributed in China, of which only *Gardenia jasminoides* is widely distributed in provinces south of the Yangtze River, while the natural distribution of the other species is extremely restricted (Li et al. 2017). *Gardenia stenophylla* Merr., 1922 is only distributed in a few areas of Guangdong, Guangxi, Hainan, and other provinces. Its plants are considered graceful with fragrant and beautiful flowers, so it is often planted as an ornamental. However, its fruits and roots are used for medicinal purposes, having cooling and detoxifying effects (Zheng et al. 2012). Notably, *G. jasminoides* is a well-known medicinal plant both domestically and internationally, while *G. stenophylla* is also used as a source of the traditional Chinese medicine Gardeniae Fructus in some provinces such as Guangdong. *G. stenophylla* displays narrow-lanceolate or linear-lanceolate leaves, which are distinctly narrower than the *G. jasminoides*. The fruit of *G. stenophylla* Merr is oblong with a diameter often less than 1.3 cm, which is also significantly smaller than that of *G. jasminoides*. When using EST-SSR to analyze the genetic relationships in *Gardenia* spp., *G. stenophylla* Merr showed a closer genetic relationship to *G. jasminoides* than the ornamental varieties of *G. jasminoides* var. fortuniana (Deng et al. 2021).

Given that *G. stenophylla* is utilized as a substitute for the traditional Chinese medicine Gardeniae Fructus in certain regions, investigating the rationality of its use as a source plant for this medicinal preparation is of significant importance. Consequently, an in-depth analysis of the phylogenetic position of *G. stenophylla* within the genus Gardenia and its genetic relationship with *G. jasminoides* has become particularly crucial. The study on the chloroplast genome sequencing and phylogenetic relationship of *G stenophylla* Merr not only provides essential reference for the selection of related species in *Gardenia* breeding but also contributes to the diversification of medicinal, ornamental, and edible plant resources within the genus. Furthermore, this research will provide scientific evidence for the taxonomic classification of Gardenia species. The outcomes of this study are expected to have substantial implications for the molecular systematics of Gardenia, conservation of genetic diversity, and the rational utilization of medicinal plant resources within this genus.

## Materials and methods

**Figure 1.**
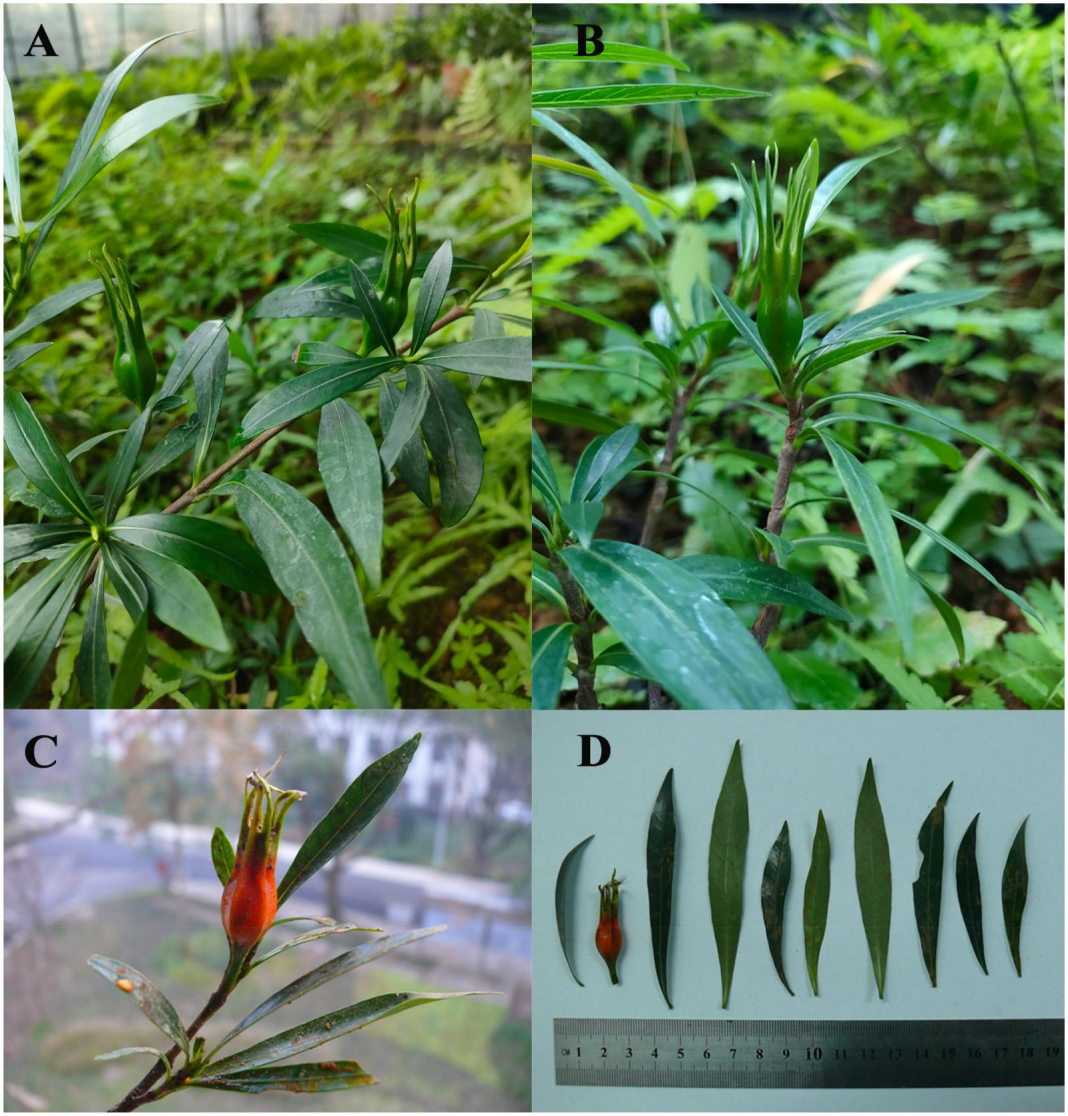
Morphological characteristics of *Gardenia stenophylla* Merr. (A–D) Photos of leaves, immature fruit, ripe fruit and obverse and reverse views of the leaves, respectively (photos taken by Shaoyong Deng in the greenhouse facility at the Jiangxi Academy of Forestry, Nanchang, China).

*Gardenia stenophylla* Merr was obtained from the Guangxi Province and planted in greenhouse conditions at the Jiangxi Academy of Forestry (Figure 1), Nanchang, China (28.74°N, 115.82°E). The voucher specimen was deposited in the Tree herbarium, Jiangxi Academy of Forestry (voucher number: XYZZ01; contact: Shaoyong Deng, jxforestry@163.com). Total genomic DNA of fresh leaves was extracted with TGuide plant genomic DNA prep kit (Tiangen Biotech, Beijing, China).

After the quality of extracted genomic DNA was verified, Illumina Hiseq 4000 (Illumina, San Diego, CA) was used for paired-end reads sequencing. A total of 102,724,070 raw reads were obtained and further filtered by NGSQCToolkit v2.3.3 (Patel and Jain 2012) to remove low-quality reads. DNA sequences were assembled *de novo* using the GetOrganelle pipeline (https://github.com/Kinggerm/GetOrganelle) and the *Gardenia jasminoides* chloroplast genome (154,921 bp, NC 057593.1) as a reference with a kmer value of 39. Annotations were generated with the annotation tools GeSeq (https://chlorobox.mpimp-golm.mpg.de/geseq.html) (Tillich et al. 2017), CPGAVAS2 (Shi et al. 2019) (http://www.herbalgenomics.org/cpgavas/), and revised as necessary using Geneious 10.2.2 (Kearse et al. 2012). Chloroplast Genome Viewer (CPGView) was used to produce the circular chloroplast genome map of *Gardenia stenophylla* Merr (Liu et al. 2023).

**Figure 2.**
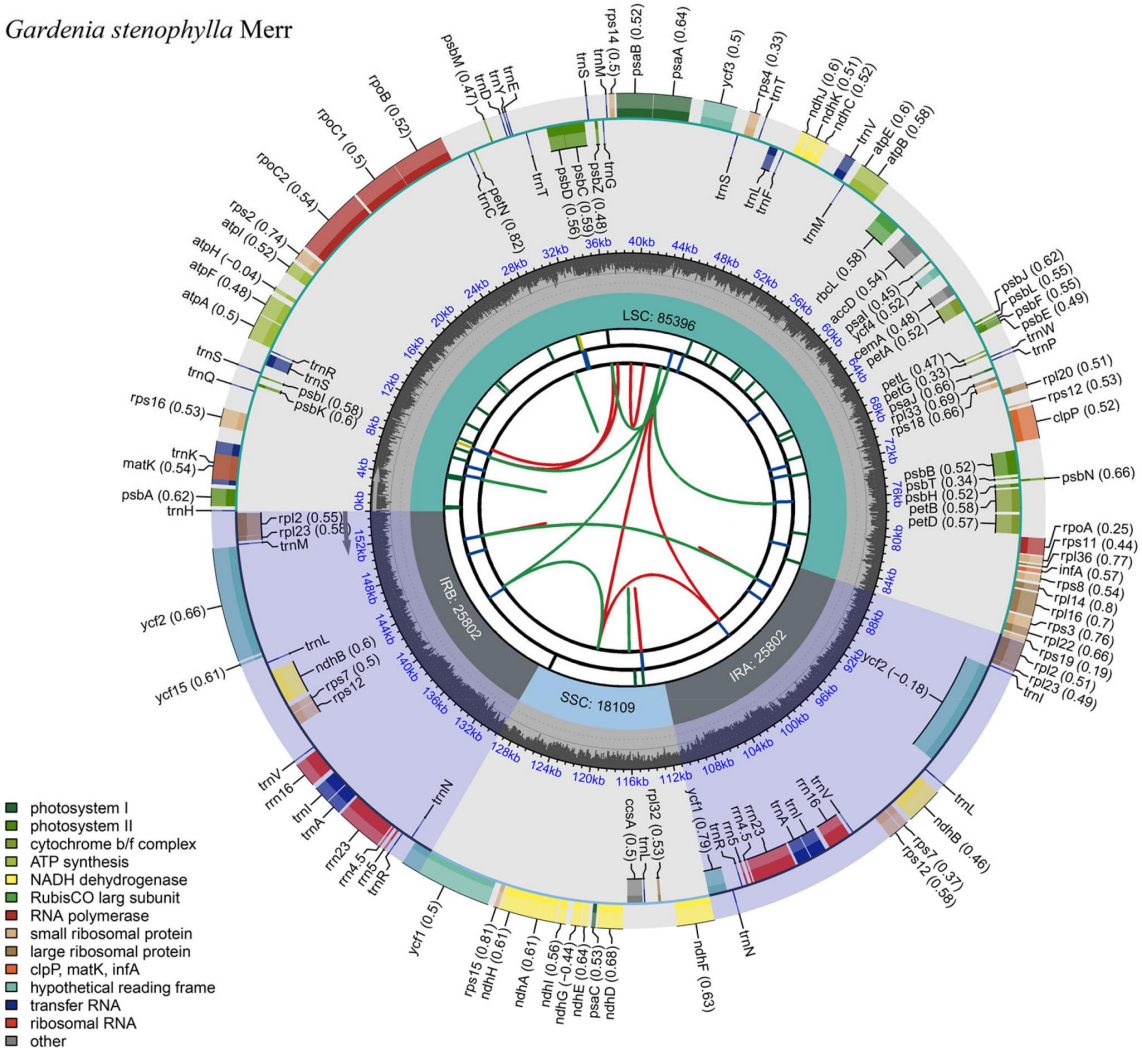
Maximum-likelihood phylogenetic tree based on 18 chloroplast genome sequences. *Gardenia stenophylla* Merr (OL517769.1) is marked in red. GenBank accession numbers are listed after their corresponding species. Bootstrap support values are indicated at each node. The following sequences were used: *Gentiana scabra* (NC 053842.1) (Liang et al. 2020), *Antirhea chinensis* (NC 044102.1) (Fan et al. 2019), *Cinchona officinalis* (MZ151891.1) (Arbizu et al. 2021), *Mitragyna speciosa* (KY085908.1) (Gitzendanner et al. 2018), *Uncaria rhynchophylla* (NC 053701.1) (Ling and Zhang 2020), *Neolamarckia macrophylla* (MN877388.1) (Shi et al. 2020), *Neolamarckia cadamba* (NC 041149.1) (Li et al. 2018), *Emmenopterys henrvi* (KY273445.1) (Duan et al. 2017), *Mussaenda hirsutula* (MK203878.1) (Wang et al. 2019), *Ixora chinensis* (MZ221832.1) (Bian and Lu 2021), *Scyphiphora hydrophyllacea* (MN390972.1), *Scyphiphora hydrophyllacea* (NC 049078.1) (Zhang et al. 2019), *Coffea arabica* (EF044213.1) (Samson et al. 2007), *Coffea canephora* (KU500324.1) (Wu et al. 2017), *Fosbergia shweliensis* (NC 050962.1), *Gardenia jasminoides* (MW160432.1) (Geng et al. 2020), and *Gardenia jasminoides* var. grandiflora (MZ151502.1) (Gong et al. 2022).

The chloroplast genome of *G. stenophylla* was phylogenetically compared with those of 16 members of the Rubiaceae family (Figure 2) downloaded from NCBI (https://www.ncbi.nlm.nih.gov/genome), using *Gentiana scabra* (NC 053842.1) as an outgroup. The 18 chloroplast genomes were aligned by MAFFT v7.520 in phylosuite (Zhang et al. 2020). A maximum-likelihood (ML) phylogenetic tree was constructed in IQTREE v 2.0.3 software (Trifinopoulos et al. 2016) with 1000 bootstrap, and the GTR model was selected as the best-fit model by PartitionFinder2 (Lanfear et al. 2017).

## Results

The total length of the chloroplast genome of *Gardenia stenophylla* Merr (GenBank accession no. OL517769) was 155,109 bp with an overall GC content of 37.5%.

To validate the accuracy of the assembled genome sequence, we mapped sequencing reads back to the assembled sequence. We achieved a depth coverage ranging from ×1388 to ×6184 across the assembled genome, with an average depth of ×3820.32 (Supplementary Figure 1). The results indicate that the assembly is reliable.

**Figure 3.**
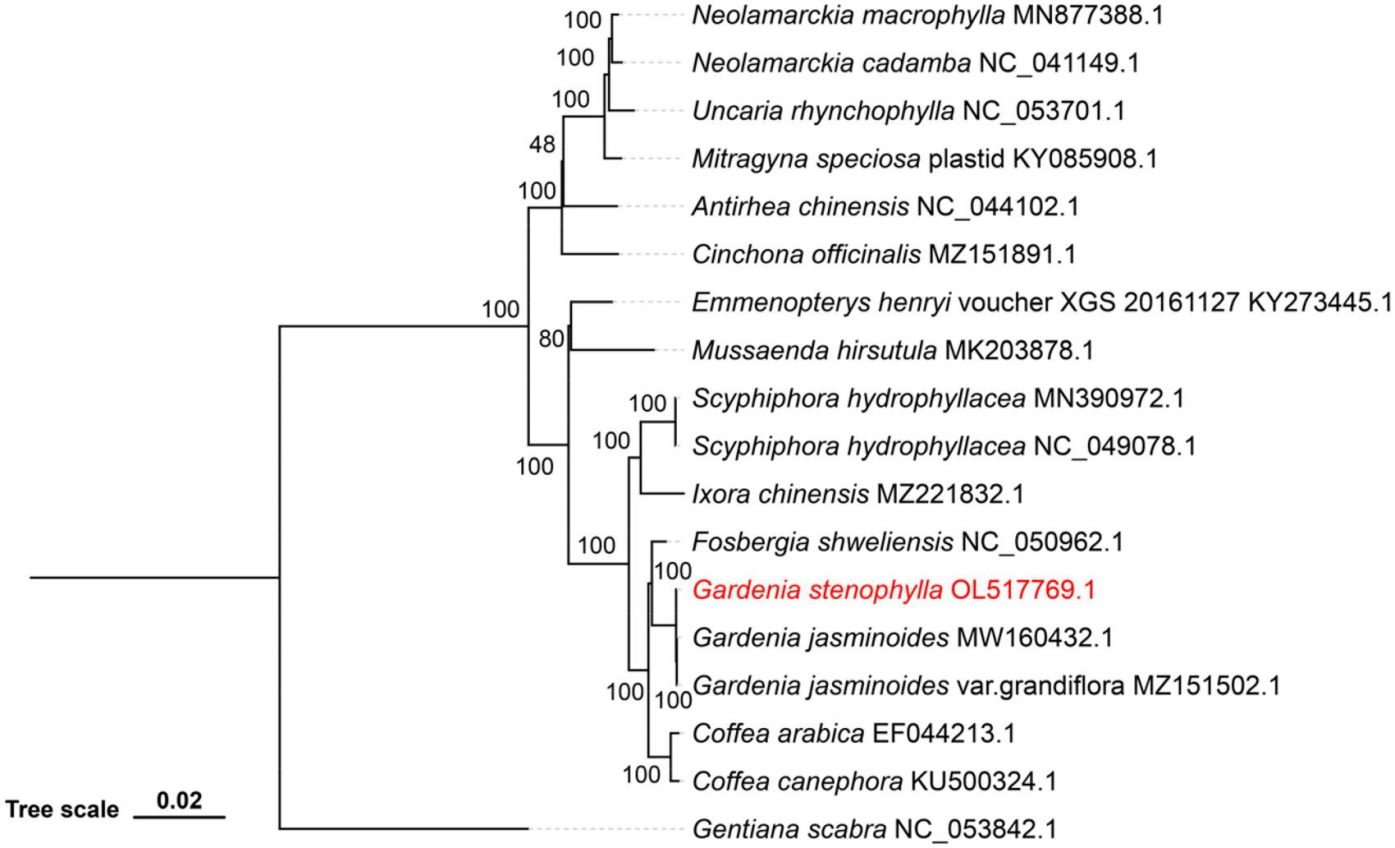
Circular map of the complete chloroplast genome of *Gardenia stenophylla* Merr. The map contains six tracks. From the center going outward, the first circle shows the distribution of sequence repeats connected by either red (the forward direction) or green (the reverse direction) arcs. The second circle shows the distribution of long tandem repeats as short blue bars. The third track shows the distribution of microsatellite sequences as short bars with different colors. The fourth circle shows the sizes of the chloroplast genome regions, including the small single-copy (SSC), inverted repeat (IRA and IRB), and large single-copy (LSC) regions. The fifth track shows the GC content along the genome. The sixth and outer circle shows the distribution of genes colored according to their functional group (legend on the left). Genes indicated inside the circle are transcribed clockwise, whereas genes indicated outside the circle are transcribed anticlockwise.

The genome showed a typical quadripartite structure, consisting of a large single-copy (LSC) region, a small single-copy (SSC) region and a region containing two inverted repeats (IRa and IRb), which were 85,396, 18,109, and 25,802 bp, respectively. The chloroplast genome contains a total of 151 genes encoding 106 proteins, 37 tRNAs, and eight rRNAs (Figure 3). Of these genes, 18 were duplicated in the IR regions, including those encoding seven proteins (*rpl2*, *rpl23*, *ycf2*, *ycf15*, *ndhB*, *rps7*, and *rps12*), seven tRNAs (*trnM*, *trnL*, *trnV*, *trnI*, *trnA*, *trnR*, and *trnN*), and four rRNAs (*rrn16*, *rrn5*, *rrn4.5*, and *rrn23*). Nine genes (*rps16*, *atpF*, *rpoC1*, *petB*, *petD*, *rpl16*, *rpl2*, *ndhB*, and *ndhA*) contained a single intron, while two genes (*ycf3* and *clpP*) contained two introns (Supplementary Figure 2).

To probe the phylogenetic relationship of *G. stenophylla* with the family Rubiaceae, a phylogenetic tree was constructed from the chloroplast genomes of *G. stenophylla*, 16 other Rubiaceae members and that of *Gentiana scabra* (Gentian family) as an outgroup. The result confirmed that *G. stenophylla* is a member of *Gardenia* and indicates that it displays the closest genetic relationship with *G. Jasminoides*.

## Discussion and conclusions

In this study, we assembled and annotated the chloroplast genome sequence of *G. stenophylla* Merr for the first time. We found that the chloroplast genome structure of *G. stenophylla* includes a pair of IRs, a SSC, and a LSC, which is consistent with the chloroplast genome structure of most angiosperms. Detailed annotation of the genome revealed protein-coding genes, tRNA genes, and rRNA genes. To determine the phylogenetic position of *G. stenophylla* within the Rubiaceae, we conducted a ML phylogenetic analysis. The results indicated that *G. stenophylla* indeed belongs to the genus *Gardenia* and shares the closest genetic relationship with *G. jasminoides*. This finding provides important insights into the evolutionary relationships within the genus *Gardenia*. Additionally, we observed that the chloroplast genome size of *G. stenophylla* does not significantly differ from those of other published Rubiaceae species (Zhang et al. 2021).

In conclusion, this study reports the complete chloroplast genome sequence of *G. stenophylla* for the first time, providing valuable foundational data for further studies in the molecular systematics, evolutionary biology, and genetic diversity of the genus *Gardenia* and the Rubiaceae family. This information serves as a crucial reference for expanding the medicinal plant resources of the genus *Gardenia* and for parent selection in the hybrid breeding of *G. jasminoides*. Moreover, it holds significant reference value for future studies in conservation genetics and molecular breeding of Rubiaceae plants.

## Supplementary Material

Supplemental material.docx

figure files.docx

## Data Availability

The genome sequence data obtained in this study are freely available in the NCBI GenBank (https://www.ncbi.nlm.nih.gov/) with the accession number, OL517769. The associated BioProject, SRA, and BioSample numbers are PRJNA1000224, SRR25458902, and SAMN36764308, respectively.
