# Supplementary material for: The complete chloroplast genome of Gardenia stenophylla Merr (Rubiaceae) and its phylogenetic analysis
Source: Mitochondrial DNA B Resour. 2024 Aug 12;9(8):1039–43. doi: 10.1080/23802359.2024.2389918 (PMC11321098; doi:10.1080/23802359.2024.2389918)
Supplement: Supplemental material.docx [file TMDN_A_2389918_SM0510.docx]

Figure captions

**Supplementary Figure 1** Coverage depth figure of the *Gardenia stenophylla* Merr chloroplast genome.

**Supplementary Figure 2** Schematic map of the cis-splicing genes and the trans-splicing gene rps12 in the *Gardenia stenophylla* Merr chloroplast genome.


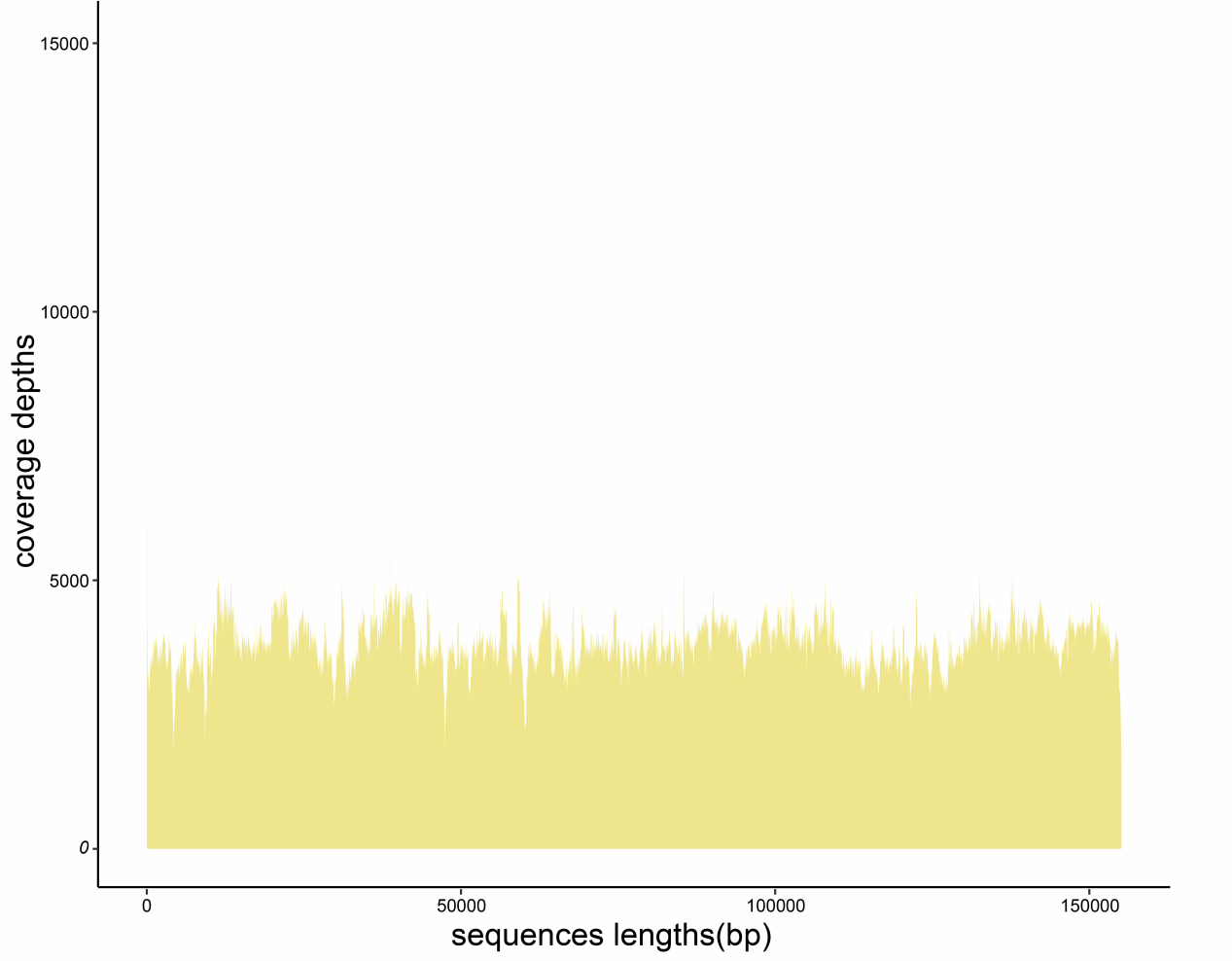


**Supplementary Figure 1** Coverage depth figure of the *Gardenia stenophylla* Merr chloroplast genome. The horizontal coordinate is the base of the chloroplast genome and the vertical coordinate is the depth of sequencing corresponding to that base.


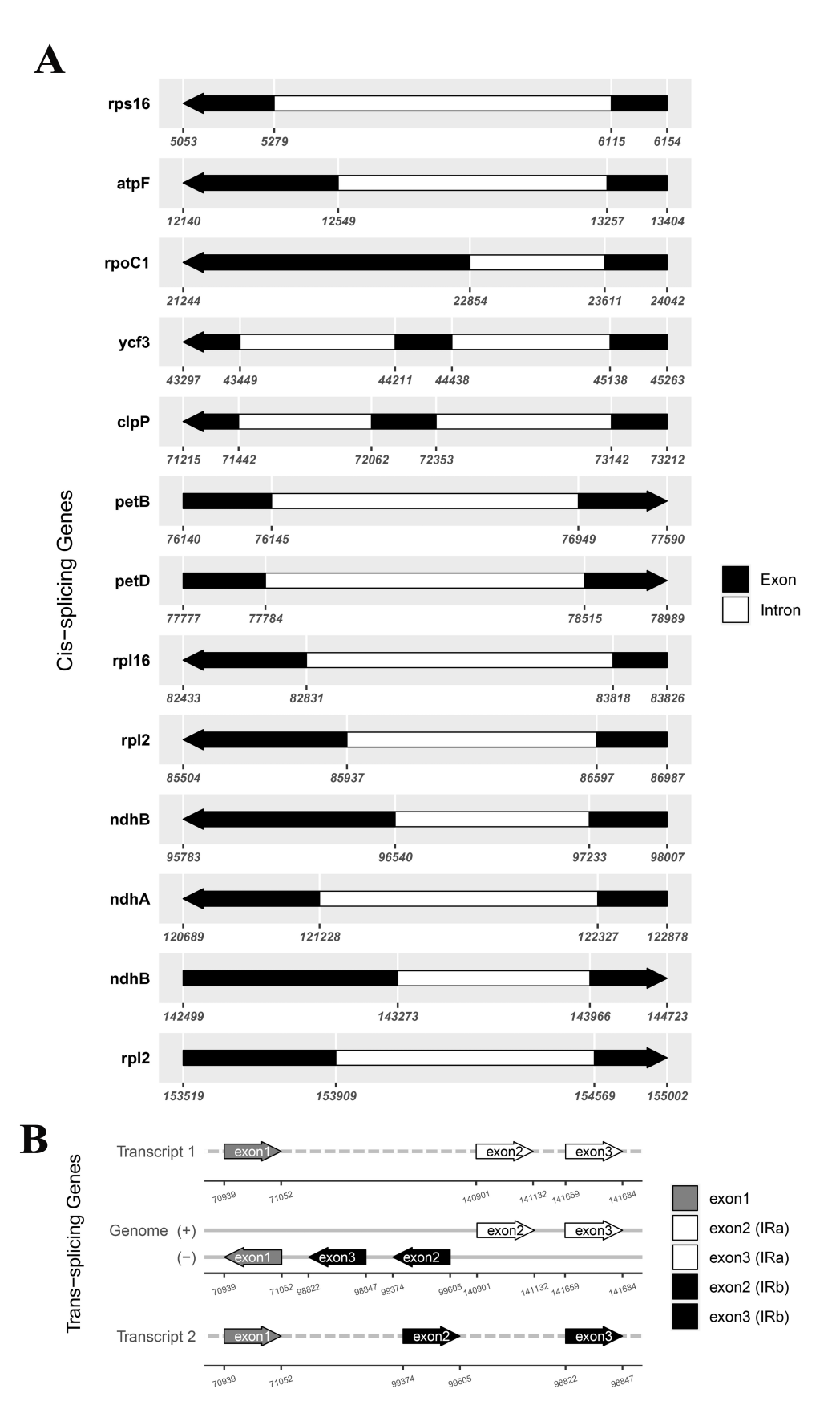


**Supplementary Figure 2** A. Schematic map of the cis-splicing genes in the *Gardenia stenophylla* Merr chloroplast genome. B. Schematic map of the trans-splicing gene rps12 in the chloroplast genome.
