# Supplementary material for: The complete chloroplast genome of Gardenia stenophylla Merr (Rubiaceae) and its phylogenetic analysis
Source: Mitochondrial DNA B Resour. 2024 Aug 12;9(8):1039–43. doi: 10.1080/23802359.2024.2389918 (PMC11321098; doi:10.1080/23802359.2024.2389918)
Supplement: figure files.docx [file TMDN_A_2389918_SM0509.docx]

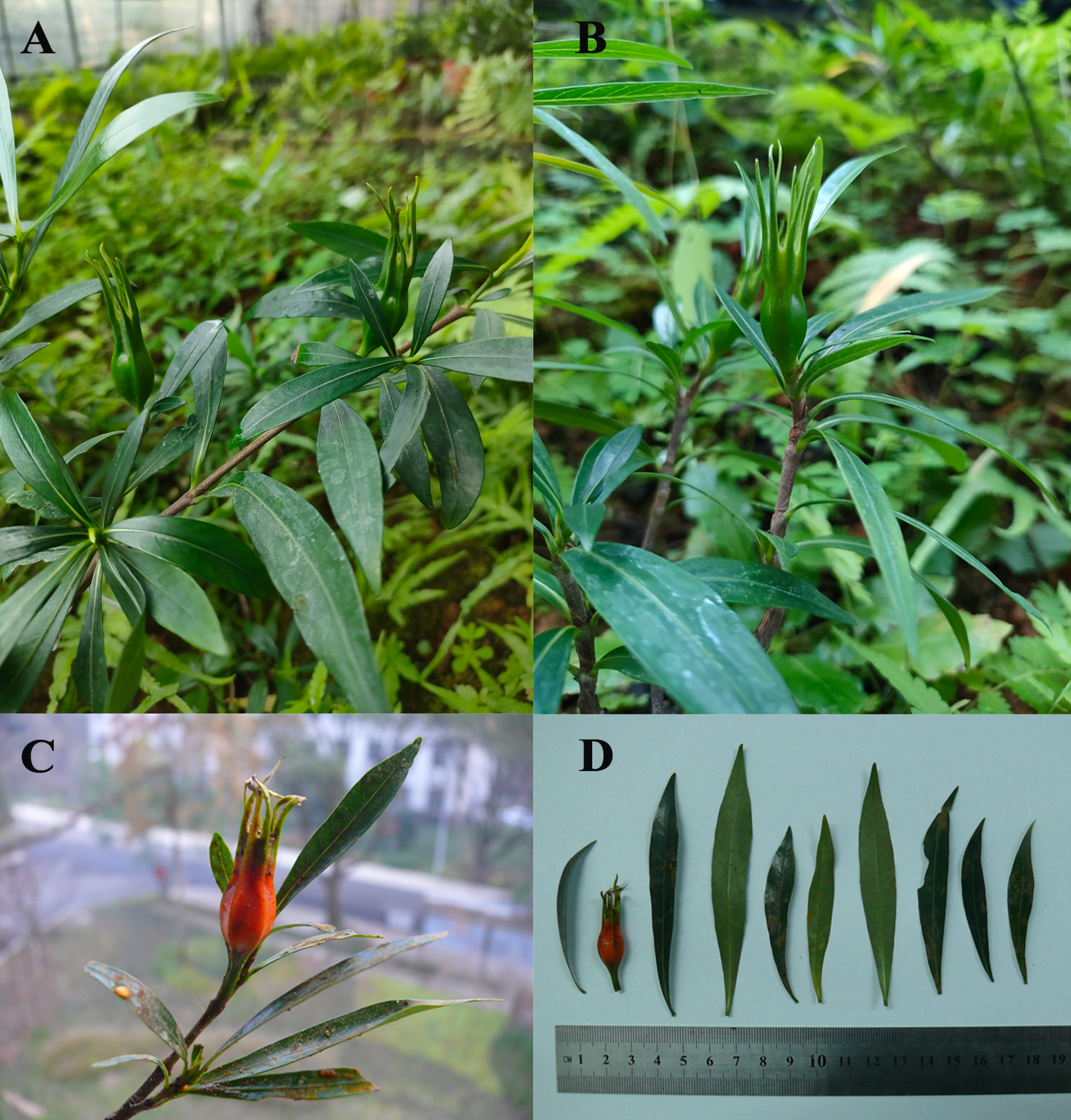


**Figure 1. Morphological characteristics of *Gardenia stenophylla* Merr**. A, B , C and D show photos of leaves, immature fruit, ripe fruit and obverse and reverse views of the leaves, respectively (photos taken by Shaoyong Deng in the greenhouse facility at the Jiangxi Academy of Forestry, Nanchang, China)





**Figure 2. Circular map of the complete chloroplast genome of *Gardenia stenophylla* Merr.** The map contains six tracks. From the center going outward, the first circle shows the distribution of sequence repeats connected by either red (the forward direction) or green (the reverse direction) arcs. The second circle shows the distribution of long tandem repeats as short blue bars. The third track shows the distribution of microsatellite sequences as short bars with different colors. The fourth circle shows the sizes of the chloroplast genome regions, including the small single-copy (SSC), inverted repeat (IRA and IRB) and large single-copy (LSC) regions. The fifth track shows the GC content along the genome. The sixth and outer circle shows the distribution of genes colored according to their functional group (legend on the left). Genes indicated inside the circle are transcribed clockwise, whereas genes indicated outside the circle are transcribed anticlockwise.


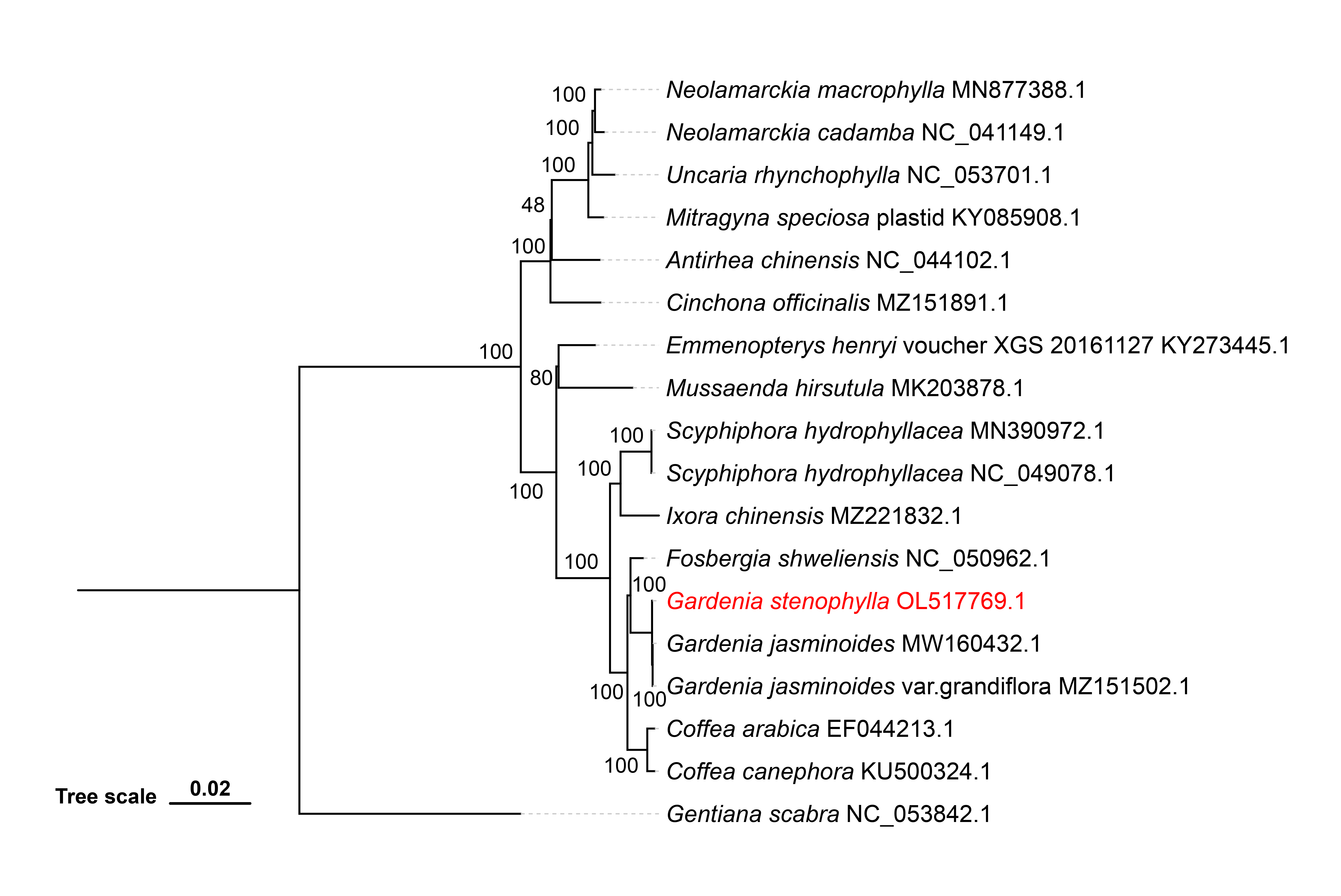


**Figure 3.** **Maximum-likelihood phylogenetic tree based on 18 chloroplast genome sequences**. *Gardenia stenophylla* Merr (OL517769.1) is marked in red. Genebank accession numbers are listed after their corresponding species. Bootstrap support values are indicated at each node.





**Supplementary Figure 1** Coverage depth figure of the *Gardenia stenophylla* Merr chloroplast genome. The horizontal coordinate is the base of the chloroplast genome and the vertical coordinate is the depth of sequencing corresponding to that base.


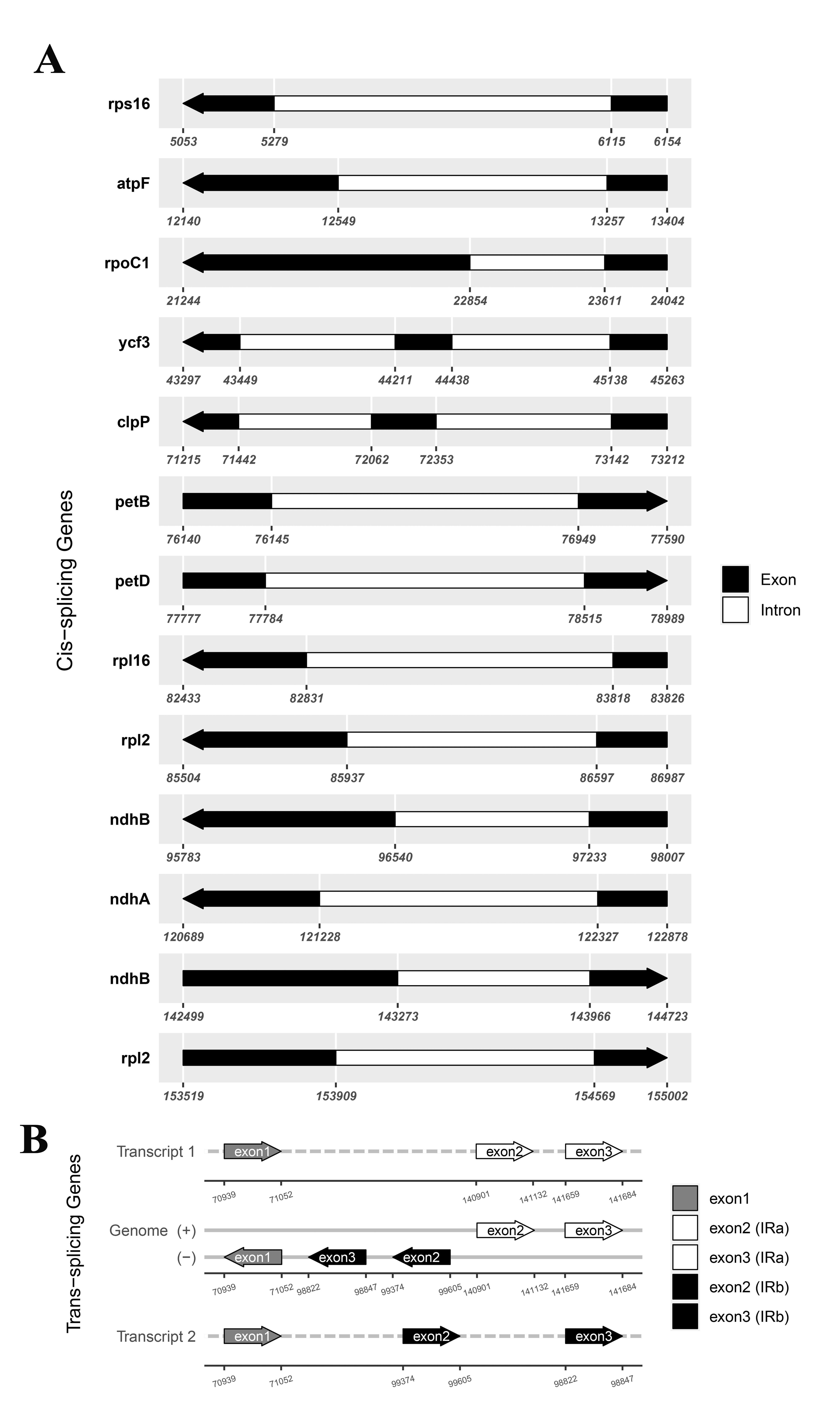


**Supplementary Figure 2** A. Schematic map of the cis-splicing genes in the *Gardenia stenophylla* Merr chloroplast genome. B. Schematic map of the trans-splicing gene rps12 in the chloroplast genome.
